# How Are Plant‐Based Milk Alternatives Presented Online? A Comparison of Information on Allergens, Nutrients and Sustainability Between Product Types From Selected UK Retailer Websites

**DOI:** 10.1002/fsn3.72169

**Published:** 2026-08-03

**Authors:** Mary Johanna Bula, Lewis W. Wallis, Helen E. Arrowsmith, Christopher A. James, Sally Moore, Pariyarath Sangeetha Thondre

**Affiliations:** ^1^ Faculty of Health, Science and Technology Oxford Brookes University Oxford UK; ^2^ School of Food Science and Nutrition University of Leeds Leeds UK; ^3^ Independent Researcher Coventry UK; ^4^ Division of Food, Nutrition and Dietetics, School of Biosciences University of Nottingham Loughborough Leicestershire UK

**Keywords:** claims, digital food environments, food hypersensitivity, nutrition, plant‐based milk alternatives, sustainability

## Abstract

Consumers in the United Kingdom (UK) are increasingly choosing plant‐based milk alternatives (PBMAs) due to food hypersensitivity, nutritional and sustainability considerations. With the growth of online shopping, comprehension of food product information available via retailer websites is important. This study evaluated allergen and nutrition information and sustainability claims for PBMAs available on three major UK retailers' websites. A review conducted between June and July 2024 collected data on 196 PBMAs from supermarket webpages, including product characteristics (processing method, sweetened/unsweetened), food allergens in the ingredients list, precautionary allergen labelling (PAL), nutrition information, claims (food hypersensitivity, environment, sustainability, nutrition and health), and the presence and type of front of pack nutrition label (FOPNL). Product types (oat, almond, soya, and coconut) and retailers were compared using one‐way ANOVA or a non‐parametric alternative test. Associations between product data and types/retailers were checked using Chi‐square tests. There was a significant association between product types and PAL (*χ*(2) = 27.651, *p* < 0.001), with PAL statements found on fewer oat (16%) and soya (14%) drinks compared to coconut (50%) and almond drinks (70%). Significant associations were identified between retailers and the presence of PAL statements (*χ*(2) = 79.268, *p* < 0.001) and hypersensitivity claims (*χ*(2) = 8.754, *p* = 0.013). The type of FOPNL used varied significantly between retailers (*χ*(2) = 12.398; *p* = 0.015), with 51%–72% products displaying no FOPNL; 16%–36% using the plain format and only 7%–13% using the Traffic Light format. These findings highlight the need for more consistent and transparent provision of information on PBMAs sold online.

## Graphical abstract

Allergen information and claims, nutrition information and nutrient profiling, dietary preference claims and sustainability claims for plant‐based milk alternatives (PBMAs).

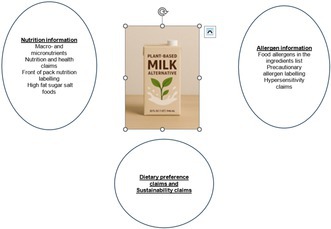

## 1 Introduction

### 1.1 Emerging Trends and Consumer Preferences for Plant‐Based Milk Alternatives (PBMAs)

Consumer preferences in the United Kingdom (UK) have significantly changed in recent years, from animal‐derived foods to plant‐based foods; a clear example is the shift from cow's milk to plant‐based milk alternatives (PBMA(s)) from sources including oat, soya, coconut, rice, almond, and other nuts (Statista 2025a). Multiple factors are driving this trend, including increasing prevalence of food hypersensitivity (comprising food allergy, coeliac disease, and food intolerance) (Hage et al. 2025), increasing concerns around animal welfare and environmental sustainability, and growing interest in the impact of diet on health (Sharma et al. 2024). Many people seek plant‐based solutions because of health conditions associated with animal‐derived milk intake; for example, milk allergy and lactose intolerance (Sethi et al. 2016). Moreover, the effects of animal husbandry on the environment are becoming more widely recognized; cow's milk production has been linked to increased greenhouse gas emissions, water usage, and land use compared to plant‐based substitutes (Xie et al. 2023). The demand for plant‐based products in all food categories, including substitutes for animal‐derived milk, has increased due to the growing popularity of vegan and vegetarian diets (Statista 2025a). This rapidly growing market for PBMAs and the range of products available poses challenges for consumers regarding differing product characteristics, making it more crucial than ever to assess the information and labelling offered on these products to ensure that consumers can make educated purchasing and consumption decisions.

### 1.2 Benefits and Challenges Associated With Nutrition and Allergen Labelling Information

Labelling PBMAs correctly is key, as information on labels must comply with relevant legislation in the jurisdiction in which the product is sold; for example, relating to food allergens, nutrition declarations, and environmental and nutrition claims (Ruby et al. 2024). PBMAs can differ greatly in terms of nutrient content depending on the base ingredient and fortification, whilst cow's milk is recognized as a source of complete protein, various vitamins and minerals (Antunes et al. 2022). Almond or rice PBMAs, for instance, may have far lower protein contents than those based on soya, which normally have a protein concentration that is comparable to that of cow's milk (Karoui and Bouaicha 2024). Some PBMAs include intentionally added micronutrients, such as calcium, iodine, and vitamin B12, which are essential for healthy bones and are naturally found in cow's milk, though the amounts may differ (Clegg et al. 2021). According to Jeske et al. (2017), appropriate fortification procedures and clear labelling are crucial to enable consumers to make knowledge‐based decisions when choosing PBMAs.

Accurate and clear nutrition labelling on PBMAs is essential for enabling consumers to make informed choices, particularly for those who rely on such products as their primary source of certain nutrients. Front‐of‐pack nutrition labelling (FOPNL), especially formats using color‐coding or warning labels highlighting nutrients of public health concern (e.g., fat, salt, and sugar), has become a powerful instrument for influencing dietary preferences and shopping habits of consumers (Song et al. 2021; Nohlen et al. 2022; Shrestha et al. 2023). This demonstrates the potential of effective product labelling to encourage healthier food choices.

The rise of online grocery shopping introduces additional challenges for food labelling and consumer information, where the presence of FOPNL can vary between product category and promotion type within online retailer websites, highlighting inconsistencies in how nutritional information is displayed online (Stones 2016; Moore and Hall 2021; Wallis and Moore 2023). In response to concerns over unhealthy diets, legislation introduced in the UK restricts the promotion, placement, and advertising of products high in fat, sugar or salt (HFSS) both in‐store and online (Department of Health and Social Care 2023). Within this policy context, it is relevant to assess the HFSS status of PBMAs sold in the UK supermarkets based on their energy, saturates, sugar, sodium, protein, fibre, fruit, vegetable, and nut content per 100 g (Department of Health and Social Care 2011).

In accordance with legislation in many regions, the labels of PBMAs must include information regarding listed substances causing food allergy or intolerance as many of these products commonly contain various food allergens including soya, almonds or oats. To protect consumers with food hypersensitivity in UK and EU jurisdictions, food information relating to substances causing allergies or intolerances present in prepacked foods should be presented in compliance with the required mandatory formats. Such information is also required to be communicated for prepacked food purchased via distance selling, for example, through websites (Council Regulation (EU) No 1169/2011 2011; Food Standards Agency 2025). However, previous research in different countries has highlighted potentially serious issues with food allergen information and labelling of food items offered for sale online, which could put consumers with food hypersensitivity at risk (Pomeranz et al. 2022; James et al. 2024; Teixeira et al. 2025). These discrepancies raise concerns about whether consumers can access sufficient and correct information when purchasing online through digital food environments.

### 1.3 Lack of Standardized Environmental Claims

Claims about sustainability and the environment have also appeared on labels of PBMAs as consumers grow more aware of environmental issues related to food products of animal origin. Such products are frequently marketed using phrases like “plant‐based”, “low carbon footprint”, “sustainable” and “good for the planet” (ASA 2024). However, confusion may result from a lack of clear definitions for these terms. Accurate and thorough labelling allows consumers to make decisions that align with their moral principles and environmental concerns (Cook et al. 2023). Given the expansion of the plant‐based and free‐from sectors and the growing popularity of online shopping, there is an urgent need to investigate current food information provision for PBMAs in the UK online retail environment. Whilst there are separate research articles published on consumer perception, nutritional, health, environmental and cost aspects of PBMAs, a comprehensive evaluation of nutrition and allergen information, and sustainability claims provided for PBMA products on the websites of UK retailers is lacking.

Therefore, the aims of this study were:

1. to describe the characteristics of different PBMA product types (oat, almond, soya, and coconut),
2. to compare product characteristics, nutrition information and labelling, food allergen information, and sustainability, nutrition and health claims between different types of PBMAs, and
3. to identify associations between retailers and nutrition information and labelling, sustainability, nutrition and health claims, food allergen information and labelling on the product information pages of PBMAs sold via three major UK retailer websites.

## 2 Methods

### 2.1 Study Design and Sample Selection

A comprehensive cross‐sectional survey examined the range of PBMAs available across three major retailers' websites in the UK between June and July 2024. The three websites selected were of the leading grocery retailers in the UK in 2024 (Statista 2025b): ASDA, Sainsbury's and Tesco labeled as R1, R2 and R3. The survey collected data for all PBMA products available on each retailer's website in the following “aisles”: *dairy and lactose‐free drinks* at R1, *plant‐based drinks* at R2 and *dairy‐free drinks* at R3.

### 2.2 Data Collection

**FIGURE 1:**
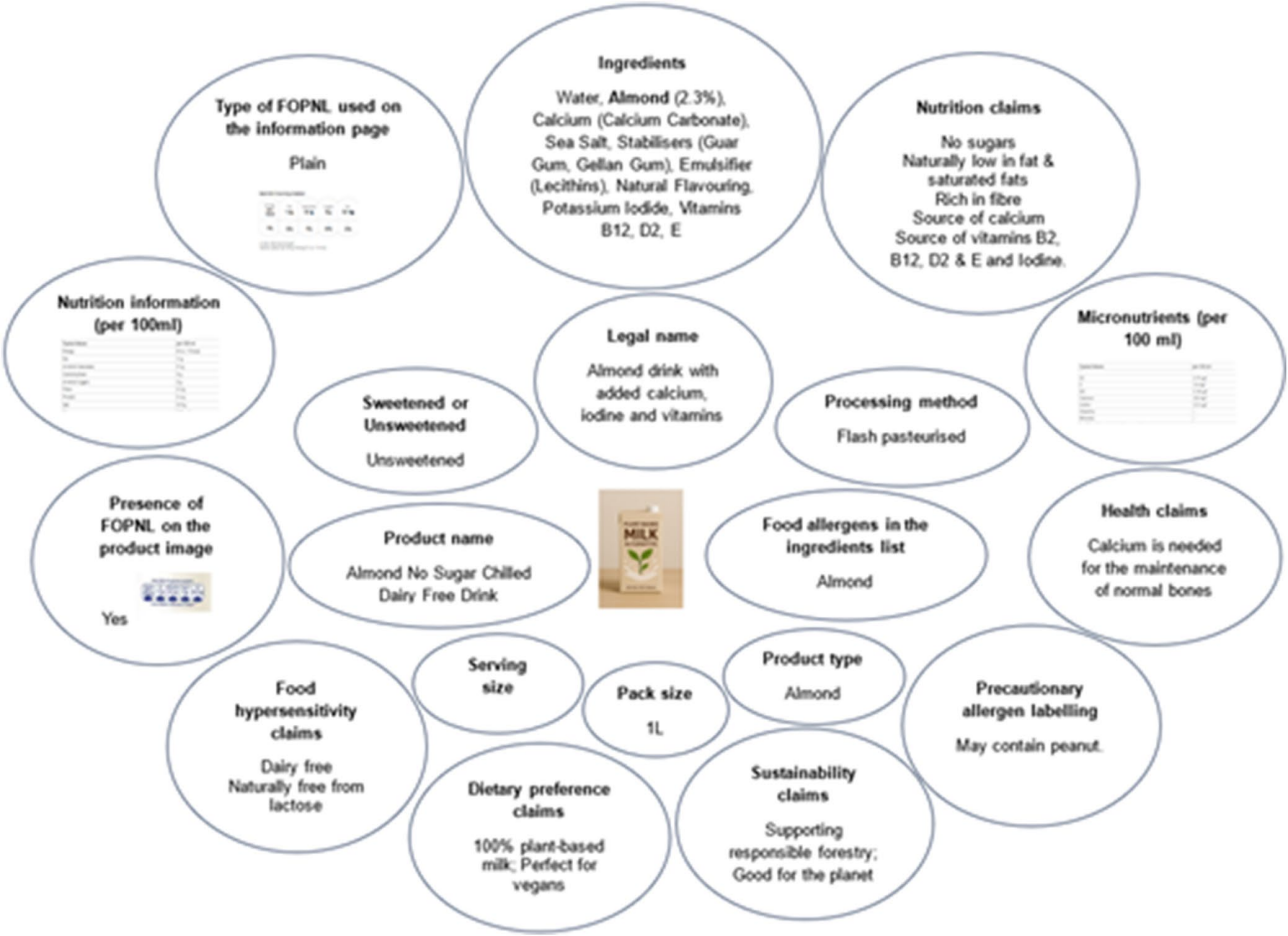
Information collected from each retailer's website shown based on an example PBMA product. FOPNL, front of pack nutrition label; PBMA, plant‐based milk alternative.

Figure 1 shows the information collected from each retailer's online platform, on a Microsoft Excel spreadsheet under separate headings, categorizing them into groups based on the primary ingredient (PBMA base). Using the Google Chrome extension GoFullPage, the product image and information including presentation, positioning, and structure of FOPNL on the webpage were recorded.

### 2.3 Data Analysis

The following data were derived based on the information collected from product information pages: nutrition information per serving (calculated from value per 100 g multiplied by serving size/100), traffic light (TL) labelling (calculated from per serving), UK nutrient profiling model (NPM) (Department of Health and Social Care 2011) score to categorize products into HFSS foods based on points for energy, sugars, saturated fat, salt, fibre (AOAC method), fruits, vegetables and nuts. PBMA drinks that scored 1 or more points were classified as HFSS products. PBMA drinks with the number of “green/amber/red” TLs were identified based on the calculated nutrition information per serving for fat, saturated fat, sugars and salt.

Oat, almond, soya and coconut drinks were included in data analysis. Other types that appeared (e.g., pea, rice, hazelnut, and cashew nut) in low numbers had data collected but were excluded because the sample size was not adequate to conduct statistical analysis. Additionally, canned coconut milk products were excluded in this study, as although they are plant‐based, they were not considered to be PBMA drinks due to their thicker consistency and predominant use in cooking. For comparisons between different PBMA product types (oat, almond, soya, and coconut), all data collected for products from the three retailers' websites were combined on one spreadsheet and the duplicates were removed in each product type before analysis.

### 2.4 Statistical Analysis

Statistical analysis was carried out using Statistical Package for the Social Sciences (SPSS) version 29.0. Descriptive statistics, including percentages, means and standard deviations, were calculated to show differences between retailers and product types. A Shapiro–Wilk test was used to check the normality of the nutrition information data; a One‐Way ANOVA test was used to analyze the normally distributed data and the Kruskal–Wallis Test was used for nonparametric data when comparing between the product types or retailers (e.g., nutrition information between oat‐, soya‐, almond‐ and coconut‐based drinks). Moreover, Pearson's chi‐squared tests were used to check the associations between the retailers or PBMA types and product information (processing methods, sweetened or unsweetened form, allergen information, PAL, food hypersensitivity claims, nutrition claims, health claims, dietary preference claims, sustainability claims, FOPNL type, and HFSS status) displayed, with statistical significance set at *p* < 0.05.

## 3 Results

### 3.1 Comparison of Product Characteristics, Allergen Information, Nutrition Information and Sustainability Claims Between PBMA Product Types and Between the Three Retailers' Websites

Data were collected from 203 PBMA products, of which 26 were coconut (13%), 30 were almond (15%), 48 were soya (24%) and 92 were oat (46%). Hazelnut (*n* = 2), pea protein (*n* = 2), rice (*n* = 2) and a cashew nut (*n* = 1) PBMAs sold via the three retailers' websites were excluded.

#### 3.1.1 Characteristics (Processing Method, Sweetened/Unsweetened) of PBMAs

Out of the 196 products, 38% (*n* = 75) had no statement of a specific processing method. The majority of products that stated a processing method (46%) used ultra‐high temperature (UHT) (R1: 50% (*n* = 33); R2: 43% (*n* = 29); and R3: 48% (*n* = 29)), whilst 17% were processed by (flash) pasteurization (R1: 13% (*n* = 9); R2: 22% (*n* = 16); R3: 15% (*n* = 9)) (Table 1). A significant association was found between PBMA product types and processing methods (*χ*(2) = 22.845, *p* = 0.007). Most oat products did not state the processing method (51%; *n* = 26/51); where it was stated, the majority were UHT processed (35%; *n* = 18), followed by flash pasteurized (8%; *n* = 4) and pasteurized products (6%; *n* = 3). Within the soya group (*n* = 29), most products were UHT processed (59%; *n* = 17) compared to flash pasteurized (28%; *n* = 8). Processing method was not mentioned for supermarket own brand soya products (10%; *n* = 3) and one branded product (3%; *n* = 1). Most products within the almond group (*n* = 20) were UHT processed (65%; *n* = 13), some were flash pasteurized (15%; *n* = 3), and others did not include the processing method (20%; *n* = 4). In the case of coconut (*n* = 20), most products did not disclose the processing method (50%; *n* = 10); those that did were mainly UHT processed (40%; *n* = 8) compared to flash pasteurized (10%; *n* = 2).

**TABLE 1:** Characteristics (types, sweetened/unsweetened, processing method) of the PBMA products sold via the three retailers' websites.

Type	Retailer	Percentage of products (*n*)^a^	Percentage of sweetened products (*n*)	Processing method of products % (*n*)
Oat	R1	47 (31)	13 (4)	UHT—48 (15) FP—7 (2) P—3 (1) No information—42 (13)
	R2	52 (36)	22 (8)	UHT—25 (9) FP—11 (4) P—8 (3) No information—53 (20)
	R3	44 (26)	8 (2)	UHT—25 (7) FP—11 (3) P—8 (2) No information—53 (14)
Soya	R1	21 (14)	71 (10)	UHT—64 (9) FP—21 (3) P—0 (0) No information—14 (2)
	R2	20 (14)	71 (10)	UHT—50 (7) FP—36 (5) P—0 (0) No information—14 (2)
	R3	27 (16)	50 (8)	UHT—69 (12) FP—19 (3) P—0 (0) No information—6 (1)
Almond	R1	15 (10)	40 (4)	UHT—70 (7) FP—20 (2) P—0 (0) No information—10 (1)
	R2	14 (10)	30 (3)	UHT—60 (6) FP—20 (2) P—0 (0) No information—20 (2)
	R3	15 (9)	11 (1)	UHT—55 (5) FP—22 (1) P—0 (0) No information—33 (3)
Coconut	R1	17 (11)	9 (1)	UHT—9 (1) FP—9 (1) P—0 No information—82 (9)
	R2	13 (9)	44 (4)	UHT—44 (4) FP—22 (2) P—0 (0) No information—33 (3)
	R3	14 (8)	25 (2)	UHT—50 (4) FP—12 (1) P—0 (0) No information—38 (3)

Abbreviations: FP, flash pasteurized; P, pasteurized; R1, retailer 1; R2, retailer 2; R3, retailer 3; UHT, ultra high temperature.

^a^ Percentages of product types reported for each retailer were calculated based on the total number of all PBMA products sold via the respective retailer's website.

The ranges of PBMAs at all three retailers included more unsweetened products (R1: 70% (*n* = 47), R2: 67% (*n* = 47), R3: 79% (*n* = 48)) compared to sweetened versions (Table 1). There was a significant association between PBMA product types and presence of added sugars (*χ*(2) = 14.389, *p* = 0.002) with more unsweetened products (78%; *n* = 40; 65%; *n* = 13; 75%; *n* = 15) within oat (*n* = 51), almond (*n* = 20), and coconut drinks (*n* = 20), respectively. Contrary to the above, there were more sweetened products (62%; *n* = 18) within the soya drinks (*n* = 29).

#### 3.1.2 Allergen Information (Intentional Food Allergens, PAL and Food Hypersensitivity Claims)

**FIGURE 2:**
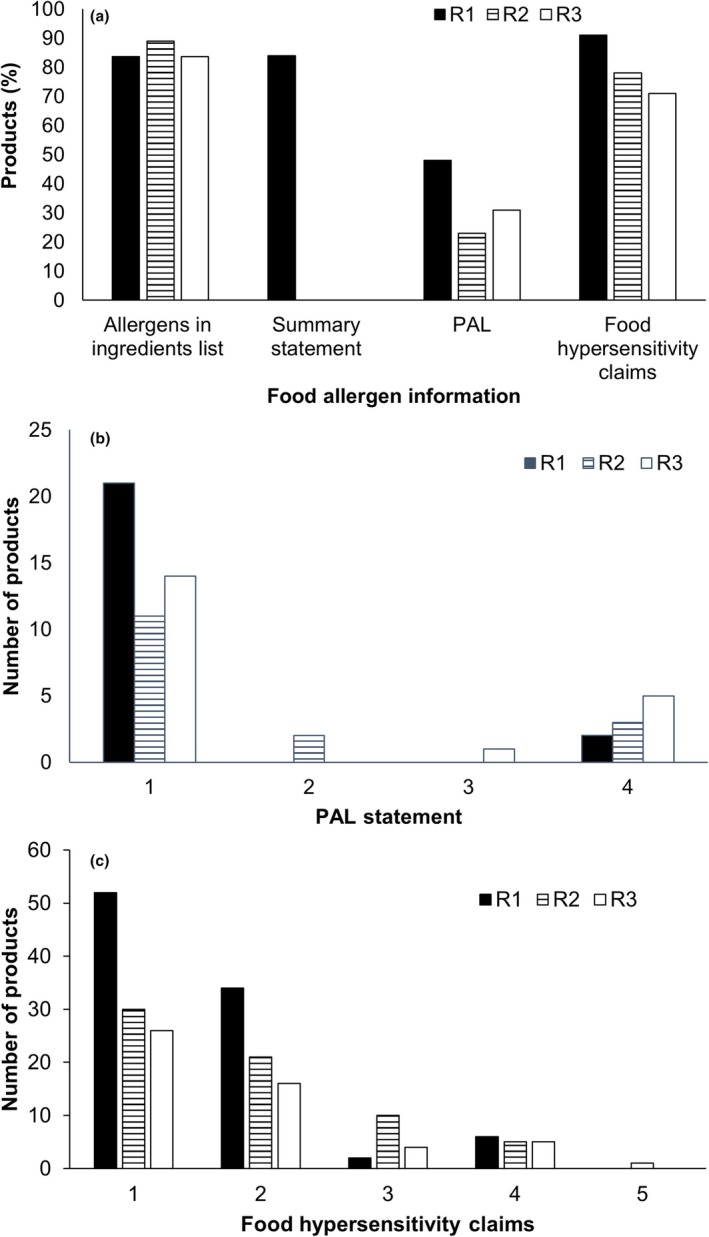
Food allergen information in different formats (a), different PAL statements (b), and food hypersensitivity claims (c) on the PBMA product pages of retailers' websites. PAL, precautionary allergen labelling; PBMA, plant‐based milk alternatives; R1, retailer 1; R2, retailer 2; R3, retailer 3.

All PBMA products intentionally containing food allergens, sold via all three retailers' websites emphasized the relevant allergens in the ingredient list on the product webpage R1: 83.6% (*n* = 56); R2: 89% (*n* = 62) and R3: 83.6% (*n* = 53), demonstrating no significant association (*χ*(2) = 1.117, *p* = 0.572). However, in addition to the food allergens emphasized in the ingredients list, R1 repeated the mandatory information using the summary statements *Contains: Oats (n = 31), Contains: Nuts (n = 3), Contains: Soya (n = 14) and Contains: Almonds (n = 10)* on 84% of the products (Figure 2a). Oat, soya and almond were the intentional food allergens listed for the PBMAs. Coconut products did not have any legislated food allergens apart from soya declared in the ingredients list of some (10%; *n* = 2/20).

A significant association was found between PBMA product types and PAL (*χ*(2) = 27.651, *p* < 0.001); very few oat (16%) and soya (14%) products had PAL statements, compared to half of the coconut (50%) and most of the almond products (70%). Within the PBMA types, the PAL statements were related to presence of nuts in the case of soya (14%; *n* = 4/29) and almond (80%; *n* = 16/20); nuts and soya in the case of coconut (50%; *n* = 10/20) and nuts, soya and gluten in the case of oat products (18%; *n* = 9/51). A statistically significant association was identified between retailers and PAL on the online product information (*χ*(2) = 79.268, *p* < 0.001) with R1 having a noticeably higher percentage (48%; *n* = 23) of products with PAL compared to R2 (23%; *n* = 16) and R3 (31%; *n* = 20) (Figure 2b). R1 displayed PAL mainly in the form of “May contain [food allergen]” statements (Table S1).

There was no significant association between PBMA types and hypersensitivity claims (*χ*(2) = 33.856, *p* = 0.277). Most of the food hypersensitivity claims within the PBMA types were to indicate that soya products were free‐from dairy/milk, gluten and lactose (79%; *n* = 23/29); almond drinks claimed to be free‐from dairy/milk, gluten, lactose, soya, eggs and peanuts (65%; *n* = 13/20); coconut drinks were stated to be free‐from dairy/milk, gluten, oats, lactose, soya, eggs, nuts and peanuts (85%; *n* = 17/20); and oat drinks were described as free‐from dairy/milk, gluten, lactose, soya and nuts (76%; *n* = 39/51). However, a significant association was found between retailers and food hypersensitivity claims (*χ*(2) = 8.754, *p* = 0.013); R1 had more products (91%; *n* = 94) with food hypersensitivity claims compared to R2 (78%; *n* = 67) and R3 (71%; *n* = 51) (Figure 2c). Most of the claims were in the form of “[food allergen] free” statements or “free‐from [food allergen]” statements.

#### 3.1.3 Nutrient Values (Macronutrients, Fibre and Micronutrients), Nutrition and Health Claims, FOPNL and HFSS Status

##### 3.1.3.1 Macronutrients, Fibre and Micronutrients

**FIGURE 3:**
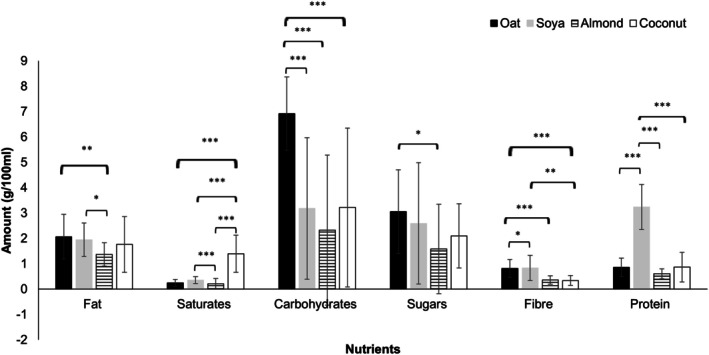
Comparison of macronutrient and fibre values (mean ± SD) displayed on PBMA product information pages. PBMA, plant‐based milk alternatives; SD, standard deviation. Significant differences are denoted by asterisks (*p < 0.05; **p < 0.01; ***p < 0.001).

There were significant differences in energy content (kcal/100 mL) of the different PBMA types (*p* < 0.001), with highest in oat (51.06 ± 9.35 kcal/100 mL; *n* = 51) followed by soya (44.57 ± 15.52 kcal/100 mL; *n* = 29), coconut (32.36 ± 14.32 kcal/100 mL; *n* = 20) and almond products (24.55 ± 13.18 kcal/100 mL; *n* = 20). Pairwise comparisons revealed significant differences between soya and almond (*p* = 0.001), oat and almond (*p* < 0.001), oat and coconut (*p* < 0.001) and soya and coconut products (*p* = 0.044). The macronutrient values of oat, nut, soya and coconut PBMAs are illustrated in Figure 3. Significant differences in fat (*p* = 0.001), saturated fat (*p* < 0.001), carbohydrate (*p* < 0.001), sugars (*p* = 0.005), fibre (*p* < 0.001) and protein (*p* < 0.001) were noted between the product types. There were no significant differences (*p* > 0.05) between the nutrient values (energy, fat, saturates, carbohydrates, sugars, protein and fibre) of PBMA products sold via the three retailers' websites (Table S2).

##### 3.1.3.2 Micronutrients

Calcium, iodine, vitamin D, vitamin B12 and riboflavin were the main micronutrients added to PBMAs. As shown in Table 2, mean calcium levels were consistent across the different PBMA types. Vitamin D levels were significantly different between the PBMA types (*p* = 0.001) with oat having higher levels compared to soya (*p* = 0.005). There was an overall significant difference in vitamin B12 levels between the PBMA types. Higher vitamin B12 content was observed in oat drinks compared to coconut drinks (*p* = 0.022). Oat, soya and almond drinks were fortified with the same amount of riboflavin compared to a higher level noted in coconut drinks. The differences were significant (*p* < 0.001 for all) between coconut and oat, coconut and soya, and coconut and nut. No significant differences were found in the mean iodine levels between the PBMA types. Only 1–5 products were fortified with folic acid, vitamin E, vitamin A, vitamin C, iron, potassium, magnesium, zinc, phosphorus, chloride and manganese (Table 2).

**TABLE 2:** Comparison of micronutrient levels (mean ± SD; per 100 mL) in PBMA products.

Micronutrients	Oat (*n*; mean ± SD)	Soya (*n*; mean ± SD)	Almond (*n*; mean ± SD)	Coconut (*n*; mean ± SD)	*p*
Vitamin D (μg)	40/51; 0.92 ± 0.20*	23/29; 0.78 ± 0.16*	9/20; 0.75 ± 0.00	14/20; 0.80 ± 0.20	0.001
Vitamin B12 (μg)	28/51; 0.42 ± 0.14*	18/29; 0.38 ± 0.00	11/20; 0.38 ± 0.00	12/20; 0.30 ± 0.10*	0.024
Riobflavin (mg)	29/51; 0.21 ± 0.00*	21/29; 0.21 ± 0.00^#^	4/20; 0.21 ± 0.00^$^	2/20; 0.50 ± 0.00*^,#,$^	< 0.001
Calcium (mg)	37/51; 116.41 ± 13.83	22/29; 120.14 ± 0.64	16/20; 123.31 ± 28.07	16/20; 125.00 ± 18.50	0.389
Iodine (μg)	28/51; 23.37 ± 4.97	13/29; 24.52 ± 4.69	7/20; 26.06 ± 6.11	3/20; 20.70 ± 13.30	0.634
Folic Acid (μg)	5/51; 15.00 ± 0.00	—	—	2/20; 40.00 ± 0.00	NA
Vitamin E (mg)	2/51; 1.55 ± 0.35	—	8/20; 1.80 ± 0.00	3/20; 3.00 ± 1.00	NA
Vitamin A (μg)	1/51; 60.00 ± 0.00	—	—	2/20; 100.00 ± 0.00	NA
Vitamin C (mg)	1/51; 12.00 ± 0.00	1/29; 12.00 ± 0.00	—	2/20; 4.30 ± 0.00	NA
Iron (mg)	1/51; 1.40 ± 0.00	1/29; 2.10 ± 0.00	—	2/20; 0.30 ± 0.00	NA
Zinc (mg)	1/51; 0.90 ± 0.00	—	—	2/20; 0.60 ± 0.00	NA
Magnesium (mg)	—	—	—	2/20; 13.00 ± 0.00	NA
Potassium (mg)	—	—	—	2/20; 83.00 ± 0.00	NA
Chloride (mg)	—	—	—	1/20; 84.00 ± 0.00	NA
Manganese (mg)	—	—	—	1/20; 0.20 ± 0.00	NA
Phosphorus (mg)	—	—	—	1/20; 78.00 ± 0.00	NA

*Note:* *n* indicates number of fortified products out of total number of products within each PBMA product type. Values with the same superscript symbol in each row are significantly different.

Abbreviations: BMA, plant‐based milk alternatives; NA, not applicable due to fortification in very few products; SD, standard deviation.

##### 3.1.3.3 Nutrition and Health Claims

**FIGURE 4:**
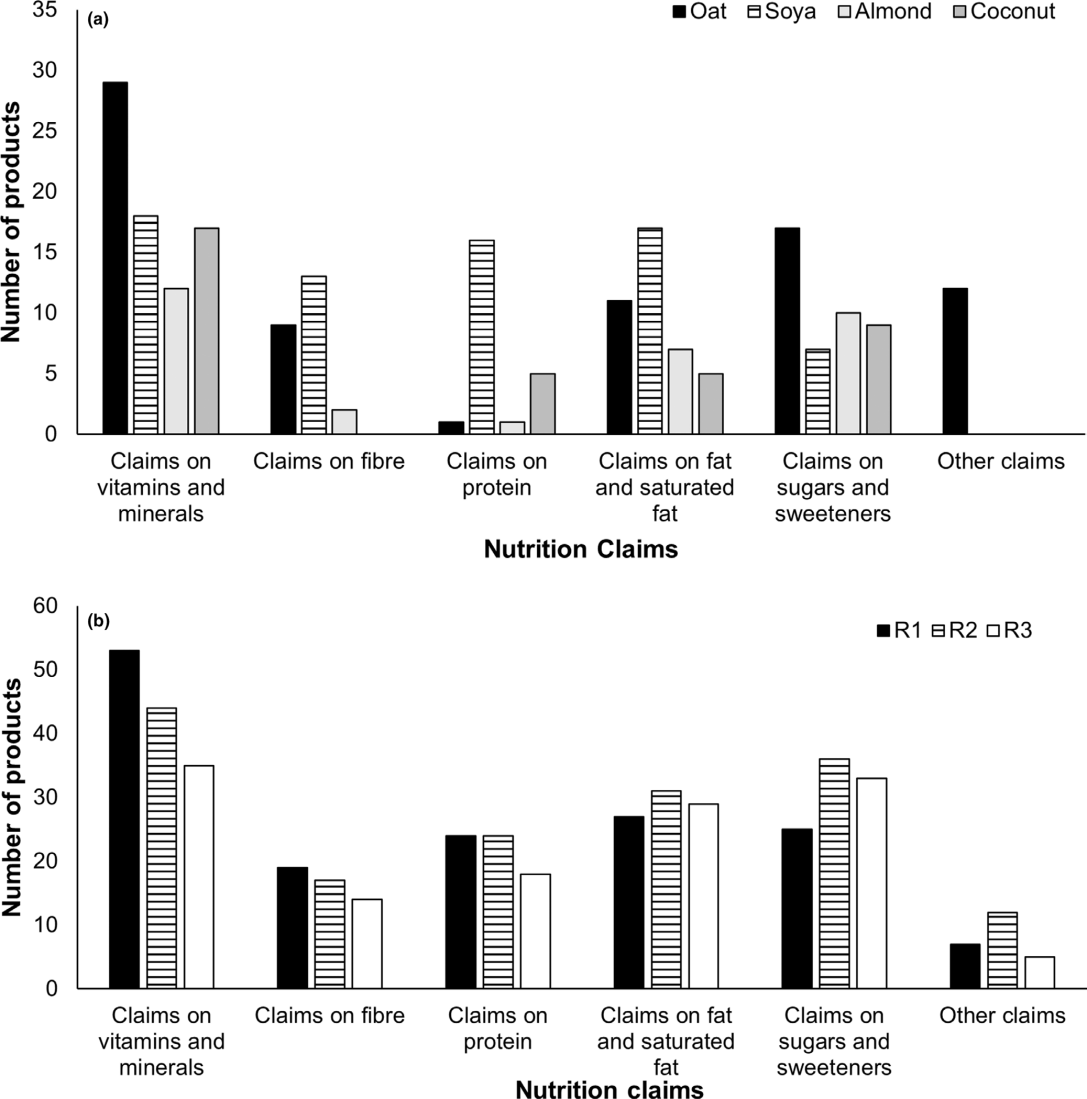
Comparison of nutrition claims (a) between PBMA product types and (b) between PBMA product pages of retailers' websites. PBMA, plant‐based milk alternatives; R1, retailer 1; R2, retailer 2; R3, retailer 3.

The association between PBMA types and display of nutrition claims on product information was not significant (*χ*(2) = 3.706, *p* = 0.295); the majority of oat (86.3%; *n* = 44), soya (79.3%; *n* = 23), almond (75%; *n* = 15), and coconut (95%; *n* = 19) drinks had one of the nutrition claims associated with fibre, macro‐ or micronutrients. Oat products had the most claims on micronutrients and sugars/sweeteners, whereas soya had the highest number of claims on protein, fibre, and fat/saturated fat. Almond and coconut had the fewest number of nutrition claims (Figure 4a). R1 had 97% (*n* = 65) of products with nutrition claims, compared to R2 (89%; *n* = 65) and R3 (74%; *n* = 45), demonstrating a significant association between retailers and display of nutrition claims on product information (*χ*(2) = 15.716, *p* < 0.001). The most prevalent claims were associated with micronutrients to indicate the product as a *source of calcium* (R1: *n* = 22; R2: *n* = 26; R3: *n* = 21) and a *source of vitamins B2, B12*, *and D(2)* (R1: *n* = 16; R2: *n* = 16; R3: *n* = 14). *Rich in fibre* (R1: *n* = 14; R2: *n* = 12; R3: *n* = 12), *naturally low in saturated fat* (R1: *n* = 10; R2: *n* = 12; R3: *n* = 10), *source of protein* (R1: *n* = 14; R2: *n* = 11; R3: *n* = 6), and *no added sugars* (R1: *n* = 9; R2: *n* = 16; R3: *n* = 14) were the next predominant nutrition claims used by the PBMA products (Figure 4b; Figure S1).

With regards to health claims and PBMA types, there was a significant association (*χ*(2) = 25.099, *p* < 0.001); the majority of oat (86.3%; *n* = 44), almond (65%; *n* = 13) and coconut (65%; *n* = 13) drinks did not use health claims, a significantly higher proportion of soya products (69%; *n* = 20) displayed health claims. The percentage of products with health claims (R1 43%; *n* = 29, R2 41%; *n* = 30, R3 43%; *n* = 26) had no significant associations with the retailers (*χ*(2) = 0.073, *p* = 0.964). The main health claims used are given in Table S3.

##### 3.1.3.4 FOPNL and HFSS Status

Fifty five percent of soya products (*n* = 16) displayed FOPNL on the product image compared to 24% of oat (*n* = 12), 30% of almond (*n* = 6) and 30% of coconut products (*n* = 6), indicating a significant association between the PBMA types and FOPNL display on packaging (*χ*(2) = 8.635; *p* = 0.035). Moreover, the type of FOPNL used on the product information page and PBMA types showed a very strong significant association (*χ*(2) = 23.537; *p* < 0.001). Whilst most of the product information pages did not show FOPNL for the oat (86.3%), almond (65%) and coconut (65%) products, a small proportion of the products used the plain format (oat: 3.9%; *n* = 2, almond: 15%; *n* = 3, coconut: 20%; *n* = 4) and the TL format (oat: 9.8%; *n* = 5, almond: 20%; *n* = 4, coconut: 15%; *n* = 3). On the contrary, an equal proportion of the products in the soya drinks displayed the FOPNL in the plain (34.5%; *n* = 10) and TL format (31%; *n* = 9) or did not display it at all (34.5%; *n* = 10).

FOPNL was clearly visible only on a small proportion of the image of the products sold by the three retailers (R1 27%; *n* = 18, R2 36%; *n* = 26, R3 25%; *n* = 15) with no significant association found (*χ*(2) = 2.248; *p* = 0.325). The type of FOPNL used on the product information page and retailers also showed a significant association (*χ*(2) = 12.398; *p* = 0.015). Whilst most of the product information pages did not show FOPNL (R1: 72%; *n* = 48, R2: 77%; *n* = 56, R3: 51%; *n* = 31), some used the plain format (R1: 16%; *n* = 11, R2: 16%; *n* = 12, R3: 36%; *n* = 22) and very few used the TL format (R1: 12%; *n* = 8, R2: 7%; *n* = 5, R3: 13%; *n* = 8) as shown in Table 3. In terms of the number of amber TLs possessed by the products (calculated using their declared amount of fat, saturated fat, sugars and salt), there was no significant association with retailers (*χ*(2) = 10.646; *p* = 0.22). Most products would require one or two amber lights on the front of pack for fat, saturates or sugars based on the calculation.

**TABLE 3:** PBMA products based on FOPNL display, possession of inherent TLs and HFSS status.

Type	Retailer	Percentage of products (*n*)	Percentage of products with FOPNL display on information page (*n*)	Percentage of products with TLs irrespective of FOPNL display (*n*)	Percentage of HFSS products (*n*)
Oat	R1	47 (31)	Not shown—84 (26) Shown—16 (5) (Plain—2; TL‐3)	1 Red—0 (0) 3 Amber—3 (1) 2 Amber—45 (14) 1 Amber—35 (11)	7 (2)
	R2	52 (36)	Not shown—92 (33) Shown—8 (3) (Plain—2; TL‐1)	1 Red—0 (0) 3 Amber—0 (0) 2 Amber—31 (11) 1 Amber—53 (19)	6 (2)
	R3	44 (26)	Not shown—77 (20) Shown—13 (6) (Plain—5; TL‐1)	1 Red—0 (0) 3 Amber—0 (0) 2 Amber—35 (9) 1 Amber—46 (12)	4 (1)
Soya	R1	21 (14)	Not shown—50 (7) Shown—50 (7) (Plain—5; TL‐2)	1 Red—0 (0) 3 Amber—0 (0) 2 Amber—29 (4) 1 Amber—50 (7)	7 (1)
	R2	20 (14)	Not shown—43 (6) Shown—57 (8) (Plain—6; TL‐2)	1 Red—0 (0) 3 Amber—0 (0) 2 Amber—21 (3) 1 Amber—64 (9)	0 (0)
	R3	27 (16)	Not shown—19 (3) Shown—81 (13) (Plain—8; TL‐5)	1 Red—0 (0) 3 Amber—0 (0) 2 Amber—25 (4) 1 Amber—63 (10)	0 (0)
Almond	R1	15 (10)	Not shown—60 (6) Shown—40 (4) (Plain—2; TL‐2)	1 Red—0 (0) 3 Amber—0 (0) 2 Amber—0 (0) 1 Amber—30 (3)	10 (1)
	R2	14 (10)	Not shown—80 (8) Shown—20 (2) (Plain—1; TL‐1)	1 Red—0 (0) 3 Amber—0 (0) 2 Amber—0 (0) 1 Amber—40 (4)	10 (1)
	R3	15 (9)	Not shown—44 (4) Shown—56 (5) (Plain—4; TL‐1)	1 Red—0 (0) 3 Amber—0 (0) 2 Amber—0 (0) 1 Amber—33 (3)	22 (2)
Coconut	R1	17 (11)	Not shown—82 (9) Shown—18 (2) (Plain—1; TL‐1)	1 Red & 1 Amber—18 (2) 3 Amber—0 (0) 2 Amber—45 (5) 1 Amber—36 (4)	73 (8)
	R2	13 (9)	Not shown—66 (6) Shown—34 (3) (Plain—2; TL‐1)	1 Red & 1 Amber—11 (1) 3 Amber—0 (0) 2 Amber—22 (2) 1 Amber—66 (6)	33 (3)
	R3	14 (8)	Not shown—25 (2) Shown—75 (6) (Plain—5; TL‐1)	1 Red—0 (0) 3 Amber—0 (0) 2 Amber—38 (3) 1 Amber—50 (4)	50 (4)

*Note:* Percentages of product types reported for each retailer were calculated based on the total number of all PBMA products sold via the respective retailer's website.

Abbreviations: FOPNL, front of pack nutrition label; FP, flash pasteurized; HFSS, High fat, sugar, salt; *n*, number of products; P, pasteurized; R1, retailer 1; R2, retailer 2; R3, retailer 3; TL, traffic light; UHT, ultra high temperature.

PBMA types and the number of products classified as HFSS showed a significant association (*χ*(2) = 24.695; *p* < 0.001). Twelve percent of oat (*n* = 6), 3% of soya (*n* = 1) and 15% of almond products (*n* = 3) belonged to the HFSS category compared to 55% (*n* = 11) of the coconut drink products. Retailers and the number of HFSS PBMA products did not show any significant association (*χ*(2) = 2.314; *p* = 0.314). The total number of HFSS products varied by retailer, of which R1 had 18% (*n* = 12) whereas R2 had 10% (*n* = 6) and R3 had 12% (*n* = 8). As shown in Table 3, these were mainly coconut, followed by oat, almond and a soya product.

#### 3.1.4 Dietary Preference Claims and Sustainability Claims

**FIGURE 5:**
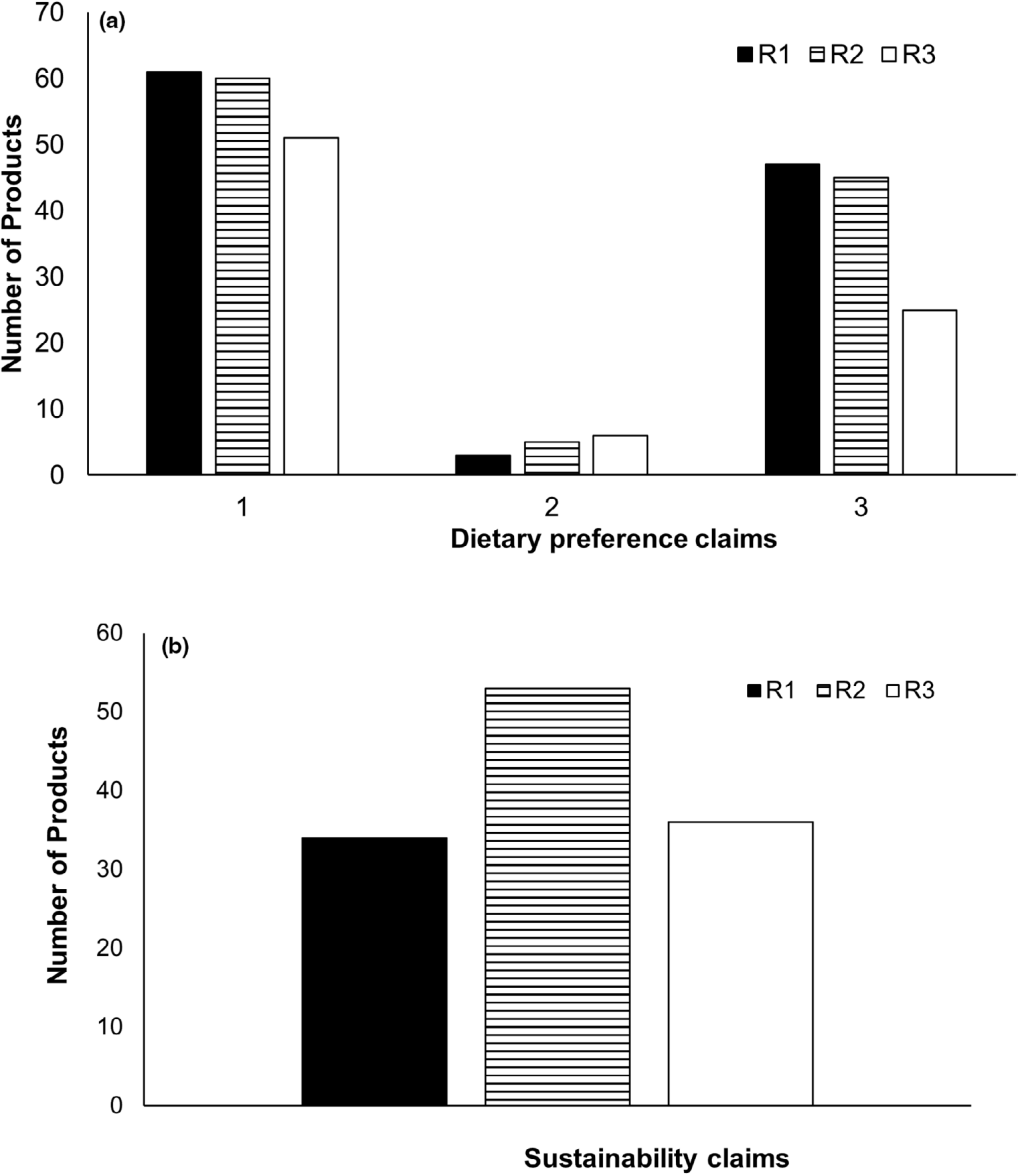
Dietary preference claims (a) and sustainability claims (b) displayed using different statements on the PBMA product pages of the retailers' websites. PBMA, plant‐based milk alternatives; R1, retailer 1; R2, retailer 2; R3, retailer 3.

Dietary preference claims were noted on the majority of the product information pages of all three retailers (R1 94%; *n* = 63, R2 96%; *n* = 66, R3 92%; *n* = 54). As seen in Figure 5a, the most used claims were to indicate the *vegan* status of the product followed by claims to show the *plant‐based* origin of the products. Very few products displayed *vegetarian* claims. In terms of sustainability claims (Figure 5b), a significant association was noted (*χ*(2) = 10.302, *p* = 0.006), with R2 having more claims on the product information page (80%; *n* = 58), followed by R1 (57%; *n* = 38) and R3 (57%; *n* = 35). *Supporting responsible forestry* was the most used claim by R1 (*n* = 26) and R2 (*n* = 35), whereas R3 mainly used the claim *Good for the planet* (*n* = 20) (Table S4).

Almost all products in each PBMA type had a dietary preference claim (oat 94%; *n* = 48, soya 97%; *n* = 28, almond 90%; *n* = 18, coconut 90%; *n* = 18) and some products had a sustainability claim (oat 62%; *n* = 32, soya 48%; *n* = 14, almond 45%; *n* = 9, coconut 30%; *n* = 6) displayed on the product information page. Therefore, there were no significant associations between the PBMA types and dietary preference claims (*χ*(2) = 1.247, *p* = 0.742) or sustainability claims (*χ*(2) = 3.817, *p* = 0.282).

## 4 Discussion

This study compared allergen and nutrition information, dietary preference, and sustainability claims across four PBMA product types (oat, soya, almond, and coconut) and examined associations between websites of three major UK retailers. The findings provide information on how PBMAs are positioned and marketed online and reveal differences in the availability and presentation of allergen, nutrition, and sustainability information and claims relevant to consumer decision‐making.

All three retailers consistently offered for sale more UHT‐processed, unsweetened PBMAs, reflecting growing consumer demand in the UK for convenient, shelf‐stable and low sugar products. However, a recent study has highlighted the potential concerns regarding the formation of harmful Maillard reaction products and inferior protein and amino acid quality in UHT‐treated PBMAs (Pucci et al. 2024), raising questions about the nutritional suitability of these products as dairy substitutes. Over a third of the PBMA products reviewed did not disclose the processing method on the retailer websites, indicating a lack of full transparency, particularly in the case of coconut drinks. Soya drinks included more sweetened products, perhaps as one of the first PBMAs developed and widely used or possibly to support more desirable sensory product qualities (e.g., masking the raw beany flavor and residual aftertaste) (Ju et al. 2021).

One of the significant findings of this study is the variation in provision of food allergen information by different retailers. Although mandatory food allergen information was consistently provided for products in accordance with UK requirements, the presence and type of PAL varied considerably between retailers. This likely reflects differences in manufacturing practices, risk assessment methodologies, risk appetite, or labelling standards between brands and ingredients of PBMAs. Inconsistent PAL can create confusion and reduce trust among consumers with food allergies, highlighting the need for additional research into industry procedures for greater clarity and possible harmonization. These results confirm the findings reported by Zurzolo et al. (2017) and James et al. (2024) about the inconsistent use of PAL and the possible effects on consumer safety. Although consumers with food allergy can be protected by warranted and well justified PAL, overuse of these statements may unfairly limit their food options and dietary intakes (Dunn Galvin et al. 2019).

Identical products were observed, with a small number of brands dominating each PBMA type across the three retailers. It is therefore not surprising that the nutrition information was similar across all the PBMAs available between the three retailers' websites. However, online presentation varied, with one retailer (R3) providing more comprehensive product information pages displaying FOPNL in plain or TL format, suggesting inconsistent labelling practices between retailers, aligning with previous evidence of variability between physical and online supermarkets (Bhatnagar et al. 2021).

One of the main findings of this study is the wide variation in nutritional composition both within and across PBMA types. Oat‐based drinks were the highest in energy due to higher contributions from fat, carbohydrate and sugar content compared to the other PBMA product types. Energy content of soya drinks followed closely and provided the highest protein levels (similar to that found in cow's milk) and contained relatively high levels of other macronutrients (fat, carbohydrate and sugars). This is consistent with earlier studies that showed soya drinks to be the closest nutritional alternative to cow's milk compared to other PBMAs in terms of protein content (Jeske et al. 2017; Chalupa‐Krebzdak et al. 2018; Vanga and Raghavan 2018; Clegg et al. 2021; Pointke et al. 2022). Coconut products had the highest saturated fat levels, which is also reflected in the HFSS status of more than half of the products in this type. Although oat and soya products had higher levels of fibre than almond and coconut products, the amounts were small overall. This variability is important for consumers who use PBMAs to replace dairy milk, which has a standardized nutritional value within specific types based on fat content (e.g., whole, semi‐skimmed and skimmed) (Antunes et al. 2022).

These variations highlight the importance of checking nutrition information of PBMAs, especially if consumers are choosing them as their primary source of protein or trying to control their energy, sugar, fat and saturated fat intake to reduce the risk of obesity and other chronic diseases (Dominguez et al. 2022). For example, individuals with type 2 diabetes are advised to control the intake of products with added sugars (Dominguez et al. 2022). In this study, only very small differences in sugar content were noted between sweetened and unsweetened types of oat and coconut drinks, which could mislead consumers. Moreover, as reported by Jeske et al. (2017), the addition of ingredients such as rice in unsweetened PBMAs may contribute to large variations in sugar content following processing, making them high glycaemic index (GI) products compared to cow's milk which has a low GI. Given that fibre is also a crucial component in reducing the risk of chronic diseases (Dominguez et al. 2022), those wishing to enhance their intake of fibre ought to select oat or soya‐based PBMAs.

Most of the PBMA types were fortified with four key micronutrients (calcium, vitamin D, vitamin B12 and iodine), although large variations were evident in the added amounts of calcium in almond products, vitamin D and vitamin B12 in oat and coconut products, and iodine in coconut products. Given that dairy products are major sources of these micronutrients except for vitamin D unless fortified, the population's intake may be significantly impacted by inconsistent fortification methods for PBMAs (Johnson et al. 2024; Clegg et al. 2021; Pointke et al. 2022). Therefore, public health authorities may want to consider mandatory fortification for PBMAs, if these products are included in the dairy category of food‐based dietary guidelines (Klapp et al. 2022). This is particularly relevant in the case of UK where there is no mandatory iodine fortification requirement and the national data has shown inadequate iodine status in women of childbearing age (16–49 years), based on the World Health Organization (WHO) criteria (Public Health England 2020; WHO 2013). Dietary changes such as replacement of animal‐derived milks with PBMAs is one of the main contributing factors for emerging iodine deficiency in the UK, which can be detrimental for foetal brain and neurological development (BDA 2018). Similarly, vitamin A is often overlooked when considering fortification of PBMAs, despite its importance in public health (The Food Foundation 2025).

Variations in the nutritional profile of certain PBMA types are reflected in the FOPNL practices on the product images and information pages of retailers' websites. All soya products in the sample except one, were classified as non‐HFSS which explains why most soya‐based PBMAs displayed FOPNL in plain or TL format. This aligns with earlier findings from Ogundijo et al. (2021) in UK food products showing that FOPNL is more frequently displayed on “healthier” and “moderately healthy” products than on “least healthy” products. Likewise, the absence of FOPNL display for HFSS vs. non‐HFSS products has been demonstrated by assessing online webpages of UK retailers (Wallis and Moore 2023). Fewer FOPNLs have been shown to be displayed online by UK supermarkets compared to their physical counterparts (Bhatnagar et al. 2021), indicating a drawback of online grocery shopping, not able to access quick nutrition information.

Dietary preference and sustainability claims were present on almost all PBMA product information pages reviewed in this study. This is not surprising considering the reduced greenhouse gas emission and biodiversity loss generally associated with plant‐based products. Although plant‐based products, such as oat drinks, are generally associated with lower environmental impact, research suggests that environmental benefits may be reduced after adjusting for nutrient content (Geburt et al. 2022). This indicates that despite their taste and popularity, most PBMAs are potentially less efficient in converting natural resources into protein sources. Springmann (2024) has reported that compared to whole and low‐fat cow's milk, the environmental impact of PBMAs was 28% for soy, 29% for oat, 33% for almond and 41% for rice. Despite their low carbon footprint, almond products are also known for their high‐water demand (Carlsson Kanyama et al. 2021; Winans et al. 2020). These differences are not immediately apparent to consumers shopping for PBMAs online. This study found that some retailers are promoting sustainability claims more than others, potentially responding to consumers' demand for more environmentally friendly products or for market advantages; sustainability claims can differentiate products in a crowded market and drive sales (Van Doorn et al. 2021). Widespread use of sustainability and dietary preference claims using different terminologies could also be attributed to the lack of regulation and standardization on such claims as reported in online supermarkets in the United States (Gerber et al. 2024).

Although previous research has compared the general food product information available in‐store compared to online, and evaluated nutrition information of PBMA types, this study extends existing published results by integrating analysis of allergen, nutrition and sustainability information across multiple PBMA types and major retailers. However, the scope was limited to three major retailers' websites and due to the limited fortification across the product sample for certain micronutrients, statistical analysis could not be carried out.

## 5 Conclusion

This study revealed the differences in allergen and nutrition information, and sustainability claims across PBMA types displayed on selected UK retailers' websites. Although all retailers and PBMA types complied with the mandatory allergen labelling requirements, discrepancies were noted in the use and display of PAL and food hypersensitivity claims between retailers. Despite the variety of PBMAs available for consumers, soya drinks remain nutritionally superior with comparable protein content to cow's milk and fewer HFSS products. Moreover, fortification practices varied between PBMAs, with differences in the levels of added Vitamin D, Vitamin B12, and Riboflavin, raising concerns about nutritional equivalence. A significant finding was the inconsistent use and display of FOPNL on online product information pages, with the majority not using FOPNL or opting for the plain instead of TL format. Overall, consumers seeking PBMAs via retailer websites need to be aware of the differences between products and inconsistencies in the information available. There is a need for more consistent PAL practices and stricter fortification policies to ensure more nutritionally equivalent and safe PBMAs for consumers.

## Author Contributions

**Mary Johanna Bula:** investigation, writing – original draft, formal analysis, data curation. **Helen E. Arrowsmith:** conceptualization, methodology, writing – review and editing, supervision, resources. **Sally Moore:** methodology. **Lewis W. Wallis:** conceptualization, methodology, supervision, writing – review and editing, resources. **Pariyarath Sangeetha Thondre:** conceptualization, methodology, data curation, formal analysis, supervision, resources, writing – original draft, writing – review and editing, project administration, investigation. **Christopher A. James:** conceptualization, writing – review and editing, supervision, resources, methodology.

## Funding

The authors have nothing to report.

## Supporting information


**Table S1:** Different types of PAL statements on PBMA products available via the retailers' websites.
**Table S2:** Nutrition information (mean ± SD; per 100 mL) of PBMA products available on the three retailers' websites.
**Table S3:** List of health claims used on the PBMA product information pages of the three retailers' websites.
**Table S4:** List of sustainability claims used on the PBMA product information pages of the three retailers' websites.
**Figure S1:** Nutrition claims displayed using different statements on the PBMA product pages of the retailers' websites. PBMA, plant‐based milk alternatives; R1, retailer 1; R2, retailer 2; R3, retailer 3.

## Data Availability

The data that support the findings of this study are available from the corresponding author upon reasonable request.
