# Supplementary material for: How Are Plant‐Based Milk Alternatives Presented Online? A Comparison of Information on Allergens, Nutrients and Sustainability Between Product Types From Selected UK Retailer Websites
Source: Food Sci Nutr. 2026 Aug 3;14(8):e72169. doi: 10.1002/fsn3.72169 (PMC13433144; doi:10.1002/fsn3.72169)
Supplement: Supplementary file 1 [file FSN3-14-e72169-s001.docx]

**Supplementary data:**

**Table S1: Different types of PAL statements on PBMA products available via the retailers’ websites**

| **PAL statement** | **Number of products displaying the PAL statement** | | |
| --- | --- | --- | --- |
|  | *R1* | *R2* | *R3* |
| May Contain: Nuts. | 11 | 0 | 0 |
| May Contain: Nuts, Soya. | 4 | 0 | 0 |
| May contain traces of Nuts [no peanuts]. | 7 | 7 | 7 |
| May also contain traces of Nuts. | 1 | 0 | 0 |
| May contain traces of other nuts | 0 | 1 | 2 |
| May contain peanuts and nuts | 0 | 0 | 2 |
| May contain peanuts and other nuts | 0 | 0 | 1 |
| May Contain: Cereals Containing Gluten. | 1 | 0 | 0 |
| May Contain: Brazil Nuts, Cashew Nuts, Hazelnuts, Macadamia Nut / Queensland Nut, Pecan Nuts, Pistachio Nuts, Walnuts; May also contain other Nuts. | 2 | 0 | 0 |
| May Contain: Brazil Nuts, Cashew Nuts. | 1 | 0 | 0 |
| May Contain: Soya. | 1 | 0 | 0 |
| May contain traces of Soya | 0 | 2 | 1 |
| Not suitable for customers with an allergy to other nuts due to manufacturing methods | 0 | 2 | 0 |
| People with severe allergies to legumes like Peanuts and Soya should be cautious when introducing pea protein into their diet because of the possibility of a pea allergy. | 0 | 0 | 1 |
| Produced in an environment that handles Nuts. | 1 | 1 | 2 |
| Produced in a facility that handles Nuts. | 0 | 0 | 1 |
| Produced in an environment that handles Nuts and Soya. | 1 | 2 | 2 |
| This product may contain traces of Soya and Tree Nuts. | 2 | 1 | 1 |
| **Total** | **32** | **16** | **20** |

PAL – precautionary allergen labelling; PBMA – plant-based milk alternatives; R1 – Retailer 1; R2 – Retailer 2; R3 – Retailer 3

**Table S2: Nutrition information (Mean ± SD; per 100 ml) of PBMA products available on the three retailers’ websites**

| Product type | Retailer | Energy (kcal) | Fat (g) | Saturates (g) | Carbohydrates (g) | Sugars (g) | Fibre (g) | Protein (g) | Salt (g) |
| --- | --- | --- | --- | --- | --- | --- | --- | --- | --- |
| Oat | R1 | 51.8 ± 9.1 | 2.14 ± 0.83 | 0.25 ± 0.15 | 6.87 ± 1.46 | 3.03 ± 1.74 | 0.83 ± 0.39 | 0.93 ± 0.34 | 0.11 ± 0.02 |
|  | R2 | 49.9 ± 10.2 | 1.99 ± 0.97 | 0.24 ± 0.11 | 6.76 ± 1.54 | 2.87 ± 1.62 | 0.84 ± 0.41 | 0.83 ± 0.39 | 0.11 ± 0.03 |
|  | R3 | 50.5 ± 9.7 | 2.08 ± 0.90 | 0.45 ± 1.05 | 6.72 ± 1.43 | 2.81 ± 1.73 | 0.81 ± 0.46 | 0.82 ± 0.43 | 0.10 ± 0.02 |
| Soya | R1 | 43.9 ± 16.3 | 1.74 ± 0.47 | 0.32 ± 0.12 | 3.73 ± 3.24 | 3.10 ± 2.72 | 0.79 ± 0.48 | 2.91 ± 0.80 | 0.09 ± 0.04 |
|  | R2 | 47.1 ± 17.0 | 2.18 ± 0.79 | 0.39 ± 0.16 | 2.98 ± 2.79 | 2.34 ± 1.99 | 0.95 ± 0.51 | 3.34 ± 1.04 | 0.11 ± 0.04 |
|  | R3 | 44.2 ± 14.0 | 1.91 ± 0.41 | 0.34 ± 0.12 | 3.08 ± 2.74 | 2.25 ± 2.18 | 0.71 ± 0.47 | 3.41 ± 0.72 | 0.11 ± 0.06 |
| Almond | R1 | 20.7 ± 6.1 | 1.36 ± 0.44 | 0.26 ± 0.31 | 1.18 ± 1.22 | 1.03 ± 1.21 | 0.36 ± 0.12 | 0.60 ± 0.21 | 0.14 ± 0.03 |
|  | R2 | 25.6 ± 13.3 | 1.37 ± 0.43 | 0.13 ± 0.07 | 2.49 ± 3.36 | 1.63 ± 2.04 | 0.38 ± 0.17 | 0.64 ± 0.19 | 0.14 ± 0.04 |
|  | R3 | 26.1 ± 18.3 | 1.28 ± 0.56 | 0.14 ± 0.10 | 2.86 ± 4.17 | 1.61 ± 2.03 | 0.36 ± 0.13 | 0.50 ± 0.20 | 0.11 ± 0.03 |
| Coconut | R1 | 29.9 ± 11.7 | 2.08 ± 1.16 | 1.71 ± 0.75 | 1.69 ± 1.15 | 1.45 ± 1.01 | 0.31 ± 0.20 | 0.93 ± 0.56 | 0.10 ± 0.03 |
|  | R2 | 31.8 ± 14.9 | 1.49 ± 1.01 | 1.12 ± 0.66 | 3.66 ± 3.05 | 2.39 ± 1.16 | 0.31 ± 0.20 | 0.87 ± 0.67 | 0.11 ± 0.02 |
|  | R3 | 28.4 ± 14.0 | 1.32 ± 0.54 | 1.12 ± 0.51 | 2.95 ± 3.75 | 1.97 ± 1.49 | 0.21 ± 0.20 | 0.63 ± 0.58 | 0.10 ± 0.03 |

PBMA – plant-based milk alternatives; R1 – Retailer 1; R2 – Retailer 2; R3 – Retailer 3; CHO – Carbohydrates.

**Table S3: List of health claims used on the PBMA product information pages of the three retailers’ websites**

| **Health claims** | **Number of products displaying the health claim** | | |
| --- | --- | --- | --- |
|  | **R1** | **R2** | **R3** |
| Calcium, vitamin D and protein are needed for normal growth and development of bone in children. | 1 | 1 | 1 |
| Calcium is needed for the maintenance of normal bones. | 10 | 11 | 12 |
| Calcium helps to maintain normal bones and teeth. | 3 | 0 | 0 |
| Calcium as part of a healthy balanced diet and lifestyle is needed for the maintenance of normal bones | 1 | 0 | 0 |
| With a balanced diet, calcium helps to maintain normal bones, muscle function and teeth | 0 | 0 | 2 |
| Protein, as part of balanced diet and lifestyle, contributes to the maintenance of muscle mass. | 10 | 9 | 5 |
| Protein contributes to the growth or maintenance of muscle mass | 0 | 0 | 1 |
| Iodine contributes to the normal growth of children. | 1 | 1 | 1 |
| Iron contributes to normal cognitive development of children | 1 | 1 | 1 |
| With added extras Calcium, Folic Acid, Potassium, Iodine, Magnesium, Zinc, Iron, Phosphorus, Vitamins A, B2, B12, D2, C and E to support children's growth, cognitive development, immunity and vision | 1 | 0 | 0 |
| Like all our oat drinks, this cold one has that good unsaturated fat that contributes to maintaining a recommended cholesterol level if you let it replace the saturated fats in your daily diet | 0 | 1 | 0 |
| Replacing saturated fats with unsaturated fats in your daily diet contributes to maintaining a recommended cholesterol level. | 0 | 1 | 1 |
| Replacing saturated fat in your diet with unsaturated fat helps maintain normal blood cholesterol levels, in combination with a varied, balanced diet and a healthy lifestyle. | 0 | 1 | 0 |
| Like all our oat drinks, whole has that good unsaturated fat that contributes to maintaining a recommended cholesterol level if you let it replace the saturated fats in your daily diet | 0 | 1 | 0 |
| Reducing consumption of saturated fat as part of a balanced diet and lifestyle can help maintain normal cholesterol levels. | 0 | 2 | 0 |
| **Total** | **28** | **29** | **24** |

PBMA – plant-based milk alternatives; R1 – Retailer 1; R2 – Retailer 2; R3 – Retailer 3

**Table S4: List of sustainability claims used on the PBMA product information pages of the three retailers’ websites**

| **Sustainability claims** | **Number of products displaying the sustainability claim** | | |
| --- | --- | --- | --- |
|  | **R1** | **R2** | **R3** |
| Made from Sustainable Sources | 2 | 1 | 0 |
| Good for the planet | 1 | 3 | 20 |
| Supporting responsible forestry | 26 | 35 | 16 |
| ProTerra Certified - Sustainability | 8 | 8 | 0 |
| Rain forest Alliance certified | 2 | 1 | 2 |
| Certified B Corporation | 8 | 16 | 0 |
| Climate footprint: 0.XX kg CO2 e per kg | 8 | 14 | 8 |
| The Cocoa Horizon Foundation certification | 1 | 0 | 0 |
| Each litre produces XX% fewer carbon emissions* than dairy milk | 1 | 3 | 1 |
| This package supports the Post Milk Generation | 0 | 1 | 1 |
| Bonsucro - Certified Sustainable Sugarcane | 0 | 1 | 1 |
| Buying this Tetra Pak® carton helps you care for the world's forests | 0 | 3 | 0 |
| Mighty for the planet | 0 | 1 | 0 |
| Carbon neutral | 0 | 0 | 2 |
| Climate neutral | 0 | 0 | 0 |
| Sustainable non-GMO Soya | 0 | 1 | 1 |
| Grown with zero deforestation | 0 | 0 | 1 |
| **Total** | **57** | **88** | **53** |

**Figure S1: Nutrition claims displayed using different statements on the PBMA product pages of the retailers’ websites.**

Number of products

| Nutrition claims | R1 | R2 | R3 |
| --- | --- | --- | --- |
| Source of Vitamin D(2) | 2 | 6 | 1 |
| Source of Fibre | 5 | 5 | 2 |
| Source of Folic Acid (Vitamin B9) | 2 | 2 |  |
| (A) Source of calcium | 24 | 26 | 21 |
| Source of vitamins C, B2, B12, D | 1 | 1 | 1 |
| Source of iron | 1 | 1 | 1 |
| Source of vitamin D2 and iodine |  | 2 | 0 |
| Source of iodine | 1 | 2 | 1 |
| (A) Source of vitamins B2, B12 and D(2) | 16 | 16 | 14 |
| Source of high quality protein | 8 | 11 | 9 |
| Sources of vitamins B2 & D(2) | 3 | 4 | 3 |
| Source of Protein | 14 | 11 | 6 |
| Source of calcium and vitamins B12 & D(2) |  | 1 | 1 |
| Source of calcium & vit. B12, D2, E |  | 1 |  |
| Source of vitamins B2, B12 , D2 & E and Iodine |  |  | 1 |
| A source of calcium, iodine and vitamins D3, B2 & B12 | 1 |  |  |
| Source of plant protein | 1 |  | 1 |
| Source of calcium, iodine, iron & zinc | 1 | 1 | 1 |
| Source of vitamins A, B2, B9, B12, D, C | 1 | 1 | 1 |
| Source of D,B12,Riboflavin,Calcium,Iodine |  | 1 |  |
| A source of Calcium, Iodine & Vitamins D & B12 | 1 | 1 | 1 |
| A source of Calcium and Plant Protein |  | 1 |  |
| Source of plant based calcium | 1 |  |  |
| Good Source of Protein |  |  | 1 |
| Added Extras Calcium, Vitamins D2 and B12 | 5 |  | 2 |
| Added Extras Calcium, folic acid, potassium, iodine, magnesium, phosphorus, iron and zinc; Vitamins A, B2, B12, C, D2 and E |  | 1 |  |
| With Added Calcium/With calcium |  | 1 | 2 |
| With added vitamins and minerals | 6 | 2 | 3 |
| With Added Calcium & Vitamin D | 1 |  |  |
| With added calcium and vitamins B2, B12 and D2 |  | 1 |  |
| With added Calcium, Vitamins E, B12, B2, D2 and Iodine |  |  | 1 |
| With added Calcium, Iodine and Vitamins B12 and D2 |  |  | 1 |
| With added Calcium and Vitamins B12, B2, D2 and Iodine |  |  | 1 |
| With Essential Vitamins and Minerals | 1 |  |  |
| Fortified With Vitamins And Minerals | 2 |  |  |
| Fortified with vitamins D and B9 (Folic Acid), as well as being a source of fibre |  | 1 |  |
| Boosted with 14 Vitamins and Minerals |  | 2 |  |
| Calcium, Vitamin B2, Vitamin D2, Vitamin B12, Iodine | 1 |  |  |
| Full of calcium, vitamins B2, D2, B12, and iodine | 7 |  |  |
| High in calcium |  | 2 |  |
| Low in fat |  | 4 | 2 |
| Low in sugars | 7 | 10 | 7 |
| Low in Saturated Fat | 1 | 4 | 3 |
| Naturally low in fat and saturated fat | 9 | 7 | 7 |
| Naturally low in saturated fat | 10 | 12 | 10 |
| Naturally low in fat | 2 | 2 | 2 |
| No added sugars | 9 | 16 | 14 |
| No sugars | 6 | 6 | 5 |
| No sugars, no sweeteners/No sugars or sweeteners | 1 | 1 | 2 |
| Rich in fibre | 14 | 12 | 12 |
| Rich in high quality protein | 1 |  | 1 |
| Rich in plant protein |  | 2 |  |
| Contains naturally occurring sugars | 1 | 2 | 3 |
| Free from sugars | 1 | 1 | 2 |
| 0.5% fat | 1 | 1 | 1 |
| 1.5% fat | 1 |  | 1 |
| extra creamy with 2.5% fat |  |  | 1 |
| 2.8% fat | 2 | 1 | 1 |
| Fat-Free | 1 |  | 1 |
| Light |  |  | 1 |
| Unsaturated fat and vitamin D2 | 1 | 1 |  |

PBMA – plant-based milk alternatives; R1 – Retailer 1; R2 – Retailer 2; R3 – Retailer 3
